# Mechanisms of Acupuncture in the Regulation of Oxidative Stress in Treating Ischemic Stroke

**DOI:** 10.1155/2020/7875396

**Published:** 2020-10-24

**Authors:** Xin-Tong Su, Lu Wang, Si-Ming Ma, Yan Cao, Na-Na Yang, Lu-Lu Lin, Marc Fisher, Jing-Wen Yang, Cun-Zhi Liu

**Affiliations:** ^1^Acupuncture Research Center, School of Acupuncture-Moxibustion and Tuina, Beijing University of Chinese Medicine, Beijing, China; ^2^Department of Acupuncture and Moxibustion, Beijing Hospital of Traditional Chinese Medicine Affiliated to Capital Medical University, Beijing, China; ^3^Department of Neurology, Beth Israel Deaconess Medical Center, Harvard Medical School, Boston, MA, USA

## Abstract

Ischemic stroke is the major type of cerebrovascular disease usually resulting in death or disability among the aging population globally. Oxidative stress has been closely linked with ischemic stroke. Disequilibrium between excessive production of reactive oxygen species (ROS) and inherent antioxidant capacity leads to subsequent oxidative damage in the pathological progression of ischemic brain injury. Acupuncture has been applied widely in treating cerebrovascular diseases from time immemorial in China. This review mainly lays stress on the evidence to illuminate the possible mechanisms of acupuncture therapy in treating ischemic stroke through regulating oxidative stress. We found that by regulating a battery of molecular signaling pathways involved in redox modulation, acupuncture not only activates the inherent antioxidant enzyme system but also inhibits the excessive generation of ROS. Acupuncture therapy possesses the potential in alleviating oxidative stress caused by cerebral ischemia, which may be linked with the neuroprotective effect of acupuncture.

## 1. Introduction

Worldwide, stroke still remains one of the leading causes of mortality and long-term disability, which causes healthcare systems a substantial financial burden [1, 2]. Globally, one in six people may suffer from a stroke within their lifetime, which causes more than 13.7 million cases each year [3]. Ischemic stroke accounts for 71% of all stroke cases [4], and the proportion in the USA is estimated to be higher, reaching approximately 85-87% [5]. Ischemic stroke is defined as the permanent infarction of brain tissues due to the abrupt loss of cerebral blood flow resulting in an impairment of normal neurologic function. Following the blockage of a cerebral artery by an embolus or in situ thrombosis and ensuing interruption of oxygen and energy supply, ischemic stroke leads to irreversible neuronal impairment and a cascade of molecular responses [3, 6]. At present, timely revascularization therapies including intravenous thrombolysis and endovascular thrombectomy are the main effective treatments in the early stage of ischemic stroke and are associated with improved neurological outcomes, which are recommended by the current clinical guidelines [7, 8]. However, they also have several limitations including the short therapeutic treatment window, rigorous eligibility criteria, and numerous contraindications [9, 10].

Cerebral ischemia and ischemia/reperfusion (I/R) induce a battery of biochemical and cellular reactions with the generation of excessive reactive oxygen species (ROS). Oxidative stress due to ROS overproduction plays an essential role in the fundamental pathologic progression of brain damage in ischemic stroke [11, 12]. Oxidative stress will occur when the inherent antioxidant potential to neutralize ROS is inadequate and fails to keep the endogenous redox balance. When oxidative stress takes place, ROS can lead to cytotoxicity through oxidative damage of lipids, proteins, and nucleic acids with harmful consequences for the structure and function of cerebral tissue [13, 14]. In ischemic stroke, oxidative stress can also cause neuronal apoptosis, activation of inflammatory signaling pathways, and impairment of the blood-brain barrier (BBB), all of which promote neurodegeneration and cell death [15–18].

Preclinical studies have unequivocally confirmed that oxidative stress injury should be a feasible therapeutic target of acute stroke, even though clinical trials yielded unfavourable results [19–21]. Acupuncture, as the main nonpharmaceutical intervention of traditional Chinese medicine (TCM), has been applied widely in China from time immemorial and has become increasingly popular worldwide, especially in treating cerebrovascular diseases [22, 23]. Meanwhile, a published systematic review and meta-analysis showed that acupuncture could reduce infarct size and improve neurological function in an experimental stroke model [24]. Introduced as a novel nonpharmacological antioxidant approach, acupuncture demonstrated a beneficial effect in modulating the redox status of the central nervous system [25]. Recently, emerging experimental studies have demonstrated that acupuncture exerts the neuroprotective role in acute stroke via its antioxidant potential [26–28]. Even so, the underlying mechanisms have not been extensively reviewed to date. The review will mainly focus on the evidence to illuminate the possible mechanisms of acupuncture therapy in treating acute cerebral ischemia through regulating oxidative stress.

## 2. Materials and Methods

We conducted this literature review by searching for mechanistic studies concerning the antioxidant effect of acupuncture therapy in experimental models of ischemic stroke. A literature search was undertaken in PubMed and Chinese National Knowledge Infrastructure (CNKI) on March 9, 2020. The following MeSH items or free words were chosen: “acupuncture,” “electroacupuncture (EA),” “manual acupuncture (MA),” “oxidative stress,” “antioxidative,” “antioxidant,” “free radicals,” “reactive oxygen species,” “reactive nitrogen species,” “redox” in combination with “stroke,” “cerebral ischemia,” and “ischemic cerebrovascular disease.” All the publication records in the last 2 decades (from January 2000 to February 2020) were collected.

A sum of 28 published articles met our inclusion criteria and were further reviewed. Most of these studies used similar animal ischemic stroke models, namely unilateral temporary middle cerebral artery occlusion (MCAO), while others employed bilateral common carotid artery occlusion (BCCAO) or microemboli injection. In these studies, acupuncture was performed either before or after the surgery. Six studies applied MA and 20 studies applied EA, while the other 2 studies applied other types of acupuncture. These studies reveal 4 major categories classifying the underlying mechanisms of acupuncture in the regulation of oxidative stress, namely, (1) activating the inherent antioxidant enzyme system, (2) inhibiting the excessive generation of ROS/RNS, (3) protecting proteins and lipids from oxidative damage, and (4) regulating the signaling pathways involved in redox modulation. The detailed characters of the included studies are shown in Tables 1, 2, 3, and 4.

## 3. Oxidative Stress and Its Detrimental Role in Ischemic Stroke

Introduced as a specific term, oxidative stress is defined as a pathologic state in which cells are subjected to excessive reactive oxygen or nitrogen species (ROS/RNS), and they cannot counterbalance the deleterious effects with the antioxidant defense system [12]. Under physiological conditions, a low level of ROS/RNS should be necessary to mediate and modulate cell signaling [29, 30]. ROS/RNS commonly results in detrimental cellular damage and tissue destruction at higher concentrations [13, 19]. Oxidative stress has been involved in the aging and pathological processes of various diseases, including atherosclerosis, hypertension, stroke, and neurodegenerative diseases [31, 32]. Particularly, oxidative stress plays a vital role in the pathogenesis of ischemic stroke and ensuing reperfusion injury, which is ascribed to the susceptibility of the brain to ROS-induced damage [33]. Hence, the specific neuroprotective treatment that can inhibit oxidative stress contributes to preventing neuronal injury associated with ischemic stroke.

**Figure 1:**
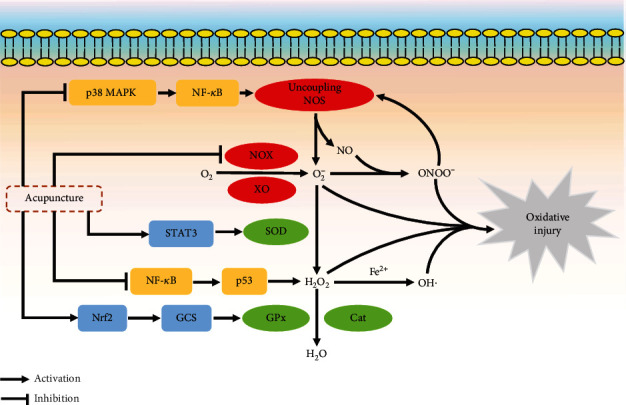
The mechanisms of ROS metabolism and the signaling pathways regulated by acupuncture in reducing oxidative stress. Cat: catalase; GCS: glutamylcysteine synthetase; GPx: glutathione peroxidase; NOS: nitric oxide synthase; MAPK: mitogen-activated protein kinase; NF-*κ*B: nuclear factor-*κ*B; NO: nitric oxide; NOX: NADPH oxidase; Nrf2: nuclear factor erythroid-2-related factor 2; STAT3: signal transducer and activator of transcription 3; SOD: superoxide dismutase; XO: xanthine oxidase.

Owing to the existence of unpaired electrons [13], ROS are short-lived and highly reactive small molecules including superoxide (O_2_^−^), hydrogen peroxide (H_2_O_2_), and the hydroxyl radical (OH·) [34] that can be produced through many pathways [35]. O_2_^−^ is the primary and initial ROS product which is formed by the univalent reduction of molecular oxygen [35]. The process is mediated via a variety of oxidative enzymes like nicotinamide adenine dinucleotide phosphate (NADPH) oxidase (NOX) and xanthine oxidase (XO) [30, 36]. Once formed, O_2_^−^ is able to produce injury directly [34]. However, under normal metabolism, the balance between superoxide production and antioxidant defense system exists, which can eliminate the overproduction of superoxide by dismutation [37]. There are three isoforms of superoxide dismutases (SODs) produced by cells that convert O_2_^−^ into H_2_O_2_: copper-zinc SOD (CuZn-SOD, SOD_1_) located in the cytoplasm, manganese SOD (Mn-SOD, SOD_2_) within the mitochondria, and extracellular SOD (EC-SOD, SOD_3_) [38]. H_2_O_2_ can be further degraded into water and oxygen via catalase or the glutathione (GSH) redox system, which consists of glutathione reductase (GR), glutathione peroxidase (GSH-Px), and peroxiredoxins (Prdxs) [19, 39]. Nonetheless, in combination with reduced transition metals such as ferrous iron (Fe^2+^) [40], H_2_O_2_ may evade enzymatic conversion and form the more highly reactive species, the hydroxyl radical [35, 41], that can cause cellular dysfunction and damage [34]. In addition to ROS, excessive O_2_^−^ can also react with nitric oxide (NO) rapidly to generate peroxynitrite (ONOO^−^) [42], which is a potent oxidizing RNS that in turn can cause oxidative injuries such as deoxyribonucleic acid (DNA) fragmentation and lipid peroxidation [43, 44]. This reaction is much more efficient than the SOD-induced dismutation of superoxide [45]. ONOO^−^ can further increase the production of O_2_^−^ by inactivating Mn-SOD and uncoupling endothelial nitric oxide synthase (eNOS), and eNOS may function as NOX to produce O_2_^−^ rather than NO [34, 35, 46]. Because of its ability to produce more superoxide, peroxynitrite might further exacerbate oxidative stress [34]. Once the redox equilibrium between the ROS production and antioxidant enzymes is impaired, it would initiate a synergistic cascade of chain reactions resulting in more secondary ROS accumulation [47, 48]. As a result, high concentrations of ROS can cause significant cellular injury in different forms [35]. A schematic illustration of the mechanisms of ROS/RNS metabolism is demonstrated in Figure 1.

Following an ischemic stroke, as a result of the sudden interruption or severe reduction of blood flow and oxygen supply to the brain, hypoxia and glucose deprivation lead to lactic acid accumulation due to energy depletion [33, 49]. Increased H^+^ concentrations provide an acid environment for promoting the prooxidant effect, enhancing the conversion rate of ROS [13, 33]. Simultaneously, an ensuing complex cascade of molecular events such as increased Ca^2+^ leads to the activation of NOX signaling and mitochondrial dysfunction, which further aggravates oxidative stress [19]. Emerging evidence has shown that elevated oxidative stress is involved in the pathophysiology of ischemic stroke and cerebral ischemia-reperfusion (I/R) injury [50–52], and inhibiting oxidative stress may play a protective role in antagonizing stroke-induced complications [18, 53]. In response to ischemic stroke, excessive ROS can cause significant cellular injury as well as vascular effects [13]. In the forms of lipid peroxidation, protein denaturation, and DNA modification as well as inducing detrimental redox-sensitive cell signaling transduction pathways [54], ROS can disrupt cellular structural integrity and cellular functions [13, 35], resulting in neuronal tissue destruction and cell apoptosis [55, 56]. High levels of ROS also have a profound vascular effect due to their hazardous effects on cerebral vascular endothelium [57]. Endothelial cell dysfunction induced by overproduction of ROS can result in disruption of the BBB [15, 16]. As a result, increased cerebrovascular permeability can further cause local edema and elevated intracranial pressure, which in turn can hinder the perfusion of the brain tissues [13, 58, 59].

## 4. Acupuncture Regulates Oxidative Stress in Ischemic Stroke

Overproduction of ROS and consequent oxidative stress have been demonstrated to exert a main role within the pathogenesis of ischemic injury [34]. The highly active metabolism of the brain asks for the constant supply of oxygen and energy. Due to the low storage of oxygen and glucose in the brain, it is especially vulnerable to the sudden interruption of cerebral blood flow. Both hypoxia-ischemia and abrupt reperfusion after ischemia can trigger the plentiful generation of ROS [6]. To date, the effects and underlying mechanisms of acupuncture involved in improving neurological outcomes of ischemic stroke have been reported by many studies. Based on these studies, the therapeutic effect of acupuncture is likely attributable to its antioxidant effect at least partially. The following 4 sections address the means of acupuncture in combating oxidative stress.

### 4.1. Acupuncture Activates the Inherent Antioxidant Enzyme System

EA therapy can significantly decrease the content of lipid peroxidation end-product malondialdehye (MDA) and 4-hydroxynonenal (4-HNE) in cerebral I/R injury rats [60–62], possibly via increasing the activities of antioxidative enzymes, namely GSH-Px and SOD [63–66]. In addition, the ginger pharma acupuncture or laser acupuncture can significantly reduce MDA production in the cerebral cortex and hippocampus while increasing catalase, GSH-Px, and SOD simultaneously [67, 68]. In multi-infarct rats, Liu et al. [69, 70] conducted two studies to explore whether acupuncture could attenuate memory impairment and ischemia-induced neuronal damage. The data suggested that acupuncture increased the expression of CuZn-SOD and a sensitive marker for oxidative injury within the hippocampus, namely redox effector factor (Ref-1), consequently producing an antioxidant effect. Consistent with the results and using the same animal model, Zhang et al. [71] found that acupuncture exerted an antioxidant effect to improve the cognitive function by upregulating the expressions of total SOD, CuZn-SOD, and Mn-SOD, as well as simultaneously elevating the ratio of reduced to oxidized glutathione (GSH/GSSG) in mitochondria. Therefore, the studies confirmed that acupuncture displayed its antioxidant properties in ischemic stroke via activating the GSH redox system and SOD-mediated dismutation. The abovementioned results are summarized in Table 1.

### 4.2. Acupuncture Inhibits the Excessive Generation of ROS/RNS

After ischemic stroke, NOX can produce a substantial amount of O_2_^−^, which represents a major and initial ROS source. NOX consists of 3 cytosolic subunits (p47phox, p67phox, and p40phox), 2 membrane subunits (p22phox and gp91phox), and the small GTPase protein [34, 72]. In the central nervous system, there are 3 isoforms of the NOX family, namely, NOX1, NOX2, and NOX4 [73]. Of note, NOX4 deletion closely correlates with reduced oxidative stress, BBB disruption, and infarct volume after MCAO [18]. A recent search designed to assess the neuroprotective effects of preischemic EA against cerebral I/R injury was conducted by Jung et al. [28]. The results revealed that EA preconditioning ameliorated neural function loss, diminished BBB permeability, and profoundly reduced NOX-derived O_2_^−^ generation through the downregulation of the expression of NOX4 but not NOX2. Furthermore, Shi et al. [74] investigated whether NOX was involved in the neuroprotection of acupuncture in a rat BCCAO model. Acupuncture dramatically decreased the O_2_^−^generation and NOX expression, as well as improved the cognitive function. In addition, a NOX antagonist mimicked the effects of acupuncture, whereas gp91phox deletion was able to reverse the antioxidant effect of acupuncture. Similarly, Guo et al. [75] also found that the decreased levels of MDA and ROS by EA pretreatment in diabetic mice with cerebral ischemia were attributable to the inhibition of NOX. Thus, acupuncture has an antioxidant effect in cerebral ischemic injury through inhibiting NOX-mediated oxidative damage, and a complete function of NOX enzyme is required in the process.

Apart from NOX-induced ROS overproduction, uncoupled NOS is another source of superoxide [46]. Moreover, NOS uncoupling can further lead to a dysregulated NO response, whereby a closely related RNS molecule—peroxynitrite—is synthesized by NO in combination with superoxide anion [19], which also exacerbates oxidative/nitrative stress injury during cerebral ischemia [43]. Inducible NOS (iNOS) expression can be upregulated by ischemia and delayed reperfusion after a stroke episode, which plays a pathological role in oxidative/nitrative stress [76]. In I/R injury rats, EA or MA effectively alleviated oxidative/nitrative stress-induced mitochondrial dysfunction and provided neuroprotection against infarct expansion by downregulating iNOS expression and peroxynitrite production, which ameliorated cognitive impairment and improved neurological function [77–79].

In addition to inhibiting the ROS-producing enzymes, it has been shown that the antioxidant properties of EA in reducing the production of ROS might be associated with the improvement of respiratory chain function in the mitochondria. Through promoting the activity of respiratory-related enzymes including cytochrome C oxidase, NADH dehydrogenase, and succinic dehydrogenase, EA remarkably improved the mitochondrial respiratory function with an ischemic state, which suggested that it could reduce the generation of ROS [80]. The abovementioned results are summarized in Table 2.

### 4.3. Acupuncture Protects Proteins and Lipids from Oxidative Damage

In the pathological progression of I/R, plenty of oxygen free radicals impair the stable equilibrium of the intracellular thiol-redox environment, leading to oxidative modification of protein thiols and dysfunction of their biological enzymatic activities such as catalytic and regulatory abilities [81]. The thiol-redox system is composed of thioredoxin, thioredoxin reductase, and NADPH, which exerts an antioxidant effect by reverting protein thiols [82]. A study indicated that EA stimulation could increase thioredoxin significantly in cerebral I/R brain tissues and reduce the oxidative stress-induced protein disulphides [83]. Of note, thioredoxin has a dual action that can keep GSH-Px fully active and increase the generation of Mn-SOD [84], which also enhances the antioxidant effect. It is desirable to explore whether crosstalk exists among the antioxidant effects mediated by acupuncture. The abovementioned results are summarized in Table 3.

### 4.4. Acupuncture Regulates the Signaling Pathways Involved in Redox Modulation

From the above studies, plenty of researchers have already explored the antioxidant effect of acupuncture in ischemic stroke. However, the precise antioxidant mechanisms of acupuncture are still necessary to be illuminated in more detail. Accordingly, it is crucial to figure out the specific molecular signaling pathways regulated by acupuncture in suppressing oxidative stress.

There exist diverse signaling pathways that participate in the pathological process of oxidative stress which have been confirmed. Therefore, the underlying signaling pathways causing ROS generation may have a close relationship with the antioxidant mechanisms of acupuncture therapy. Under cerebral I/R pathological conditions, the accumulation of damaged mitochondria becomes a major source of toxic ROS [85]. The Pink1/Parkin pathway is an important pathway in regulating mitophagy clearance, which can maintain neuronal mitochondrial quality [86]. By activating Pink1/Parkin-mediated mitophagy clearance, EA could decrease dysfunctional mitochondria and ROS production in transient MCAO models [77]. Moreover, EA also downregulated the level of iNOS via regulating the p38 mitogen-activated protein kinase (MAPK)/nuclear factor-*κ*B (NF-*κ*B) signaling pathway, which contributed salutary effects to the attenuation of nitrative stress as well as inflammation after cerebral I/R [79]. A recently published study found that acupuncture reduced the intracellular OH· generation by inhibiting the activation of p53, which is the downstream target gene of NF-*κ*B [27]. Furthermore, enhancing the activity of the parasympathetic nervous system was found to have conducive effects in cerebral ischemia [87]. Muscarinic receptors are involved in the modulation of the redox state, especially M_2_ and M_3_ receptors [88]. EA was able to upregulate 5 types of muscarinic receptors, as well as activate the muscarinic receptor-mediated parasympathetic network in the brain after ischemic stroke, which eventually reduced the level of MDA [62]. Further studies to explore the specific muscarinic receptor-mediated signaling pathway that facilitates the antioxidant effect of acupuncture are required.

Previous studies have suggested that acupuncture therapy can also promote an antioxidant effect by enhancing the function of endogenous enzymatic defenses against oxidative stress. Under normal conditions, the signal transducer and activator of transcription 3 (STAT3) has been confirmed as a transcription factor which can regulate the Mn-SOD gene expression [89]. Through the activation of cannabinoid receptor type 1 (CB1R-) mediated STAT3 phosphorylation at Y705, EA could attenuate the transient ischemic oxidative damage by upregulating the Mn-SOD protein expression and Mn-SOD-positive neuronal cells [90]. Additionally, nuclear factor erythroid-2-related factor 2 (Nrf2), as a master regulator of endogenous antioxidant genes, exerts the critical cytoprotective effect in attenuating oxidative stress [91]. Substantially restored levels of the GSH redox system by EA in cerebral ischemia might be attributed to the activation of the extracellular regulated kinase 1/2 (ERK1/2-) mediated Nrf2 signaling pathway [92]. Glutamylcysteine synthetase (GCS), which catalyzes the rate-limiting biosynthesis of GSH, is regulated by Nrf2 [93]. Several studies investigating neuroprotection against I/R injury of EA suggested that EA activated the Nrf2/GCS signaling pathway and subsequently promoted the expression of GSH remarkably [94–97]. Furthermore, the antioxidant potential of acupuncture may also be associated with the transient receptor potential vanilloid 1 (TRPV-1-) mediated signaling pathway. EA pretreatment could suppress TRPV-1-mediated p38 MAPK activation, which upregulated the expression of SOD and GSH simultaneously [26]. The abovementioned results are summarized in Table 4. A schematic illustration of the signaling pathways regulated by acupuncture in reducing oxidative stress is demonstrated in Figure 1.

## 5. Discussion

As an age-old treatment, acupuncture has been demonstrated beneficial effects for recovery from ischemic stroke, perhaps through reducing oxidative stress. According to all the convincing evidences from the studies we have reviewed, the acupuncture-induced antioxidant effect is mainly produced by enhancing the activity of antioxidative enzymes, as well as suppressing the expression of prooxidant enzymes and inhibiting the oxidative modification of cellular molecules. Modulating the molecular signaling pathways related to the generation or elimination of ROS has been suggested to be closely associated with the antioxidant effect of acupuncture. In addition, acupuncture was also found to improve the mitochondrial respiratory function and mitophagy clearance to decrease ROS production indirectly.

### 5.1. Factors Associated with the Antioxidant Effect of Acupuncture

Different from other pharmacological therapies, acupuncture is one kind of intricate intervention whose therapeutic effects are influenced by a series of factors [98]. Intervention time, type of acupuncture, and acupoint selection or combination may have an impact on the ability of acupuncture to counteract oxidative stress. The results from the preclinical studies indicate that both acupuncture pretreatment and posttreatment are able to alleviate oxidative stress after cerebral ischemia. Of note, a study further evaluated the antioxidant effect against cerebral I/R injury of the EA pretreatment group and posttreatment group in the same experiment, which found that both groups showed preferable results compared with the MCAO control group. It indicates that acupuncture pretreatment might enhance tolerance against ischemic brain insult and provide an excellent antioxidant effect as well as acupuncture posttreatment [94]. In addition, more than half of the researchers employed EA, which is a combination of modern electrical stimulation and acupuncture, while others applied MA. Despite all studies have demonstrated that both MA and EA are beneficial to attenuating oxidative stress, but neither approach has been highlighted as more effective than the other.

Although the acupoint selection varied among the included studies, the following acupoints were applied more frequently by researchers: (1) Baihui (GV20), (2) Dazhui (GV14), and (3) Zusanli (ST36). Thereinto, 70.8% of the studies employed Baihui. A summary of acupoints selected in the reviewed literatures is shown in Table 5. Both of Baihui and Dazhui, located on the Governor vessel, are the meeting points of all *yang* meridians, which can eliminate interior wind, tonify *yang*, and promote resuscitation on the view of traditional Chinese medical meridian theory [99]. From the historical perspective, Baihui is the most often-chosen principle acupoint in treating neurological diseases like ischemic stroke and dementia. Nowadays, various basic studies have also confirmed that scalp acupuncture at Baihui plays multiple neuroprotective roles including antiapoptosis, anti-inflammatory, and perfusion augmentation in the pathophysiological process of laboratory models of ischemic stroke, not only regulating oxidative stress [24, 100–102]. It has also been reported that acupuncture at Dazhui could enhance the activity of SOD and GSH-Px [78, 92], improve perfusion in the ischemic zone, and promote the proliferation of neuronal stem cells [101, 103]. Zusanli, as a well-known acupoint good at strengthening the body resistance to eliminate pathogenic factors, could exert multiple neuroprotective roles in the recovery process of ischemic stroke. Acupuncture at Zusanli was found to ameliorate oxidative damage via inhibition of NOX-mediated ROS generation [74], as well as have an antiapoptotic effect and modulate the expression of anti-inflammatory mediators [104, 105]. Moreover, although Baihui plus Dazhui is the most common acupoint combination in this review, which appeared in 29.2% of the studies, there is still no universal standard for the combination of acupoints in the antioxidant studies of acupuncture in treating ischemic stroke. Most of the researchers often determined the acupoint combination according to their own clinical experience. It is necessary to explore the optimum acupoint combination in future studies.

### 5.2. Prospects of Acupuncture as an Antioxidant Therapy for Future Studies

Generally speaking, oxidative stress is a pivotal mechanism closely relevant to early stroke treatment [6]. However, from the clinical standpoint, acupuncture is universally utilized in poststroke rehabilitation at present, which has been adopted by the National Institutes of Health [106]. In fact, acupuncture is likely to have beneficial effects in the acute treatment of cerebrovascular disease as well since ancient times. A famous classical TCM book written by Hong Ge in the Eastern Jin Dynasty (317-420 AD) named Zhouhou Beiji Fang (*Handbook of Prescriptions for Emergency*), which gave the Nobel Laureate Youyou Tu a novel inspiration of discovering artemisinin in treating malaria [107], strongly recommended acupuncture as a viable first-aid intervention in the early phase of acute stroke. Nonetheless, to date, convincing evidence from clinical trials supporting acupuncture in acute treatment for ischemic stroke may be still insufficient due to the poor methodological quality of current studies [22]. Despite the achievements in animal studies of acupuncture in the regulation of oxidative stress suggest that acupuncture may be helpful in the acute phase, the obstacles hindering successful translation from animal to clinic need to be overcome.

Acupuncture is a novel concept of therapeutically manipulating the redox state via nondrug afferent stimuli [25]. The specific significance of acupuncture as an antioxidant therapy for future clinical research needs to be elaborated. On the one hand, it seems that the antioxidant effect of acupuncture is multifaceted and a common result of different mechanisms, which shows the superiority of acupuncture compared with the single therapeutic target interventions. It may be difficult for us in determining the primary one that the antioxidant effect induced by acupuncture should ascribe to. However, it is not easy to assess the specific oxidative status of one individual patient and determine the most suitable antioxidant agent for that patient either [108], which may make sense in demonstrating the therapeutic efficacy in the current clinical trials of antioxidant interventions for acute ischemic stroke. Therefore, whether acupuncture, as a multiple-target antioxidant therapy in ischemia stroke, can achieve favorable results in clinical studies requires further investigation. On the other hand, limited by the narrow therapeutic treatment window and rigorous contraindications, under clinical conditions, not all patients can always achieve early initiation of revascularization therapy like thrombolysis or thrombectomy [3, 6], even if successful reperfusion after getting these therapeutic modalities may bring the so-called I/R injury, which is likely to be mainly attributable to overproduced ROS [109]. Acupuncture can afford an antioxidant effect but does not have a risk of increasing hemorrhagic transformation and plenty of contraindications. Hence, acupuncture stands a chance of serving as one of the potential complementary approaches for neuroprotection against acute stroke, especially in the early stage.

Several acknowledged limitations exist in our review, which can be explained as follows: (1) All the studies reviewed were conducted using animal models. However, the experimental stroke models cannot be exactly in keeping with the clinical conditions, and the differences between rodent and human brains may influence the extrapolation of the results, such as different antioxidant enzyme concentrations or reactions to acupuncture. More clinical data are needed to determine whether acupuncture is a realistic antioxidant option. (2) Although acupoint specificity remains a crucial matter to the effect of acupuncture, only a few studies set nonacupoint acupuncture or sham acupuncture as a control group in their experiments, which makes it difficult to verify acupoint specificity and the real effect of acupuncture. (3) The number of articles related to our topic and the results from these studies are limited, so the conclusions may be potentially inadequate. Moreover, the quality of some selected studies is not high enough, which may inhibit the reliability of this review.

Even though many advances have been achieved, for further studies, there are still two issues that need to be explored. Firstly, the definite mechanism concerning how the stimulation signal of acupuncture is delivered from peripheral acupoints to the central nervous system so as to exert antioxidant effect remains to be elucidated. Secondly, plenty of variables are present in the current acupuncture-related studies. Researchers need to take into consideration of comparing the antioxidant effects among different types of acupuncture and different acupuncture parameters.

## 6. Conclusions

In summary, by regulating a battery of molecular signaling pathways involved in redox modulation, acupuncture not only activates the inherent antioxidant enzyme system but also inhibits the excessive generation of ROS. Acupuncture therapy possesses the potential in alleviating oxidative stress caused by cerebral ischemia, which may be linked with the neuroprotective effect of acupuncture.

## Figures and Tables

**Table 1:** Acupuncture activates the inherent antioxidant enzyme system.

References	Species/model	Pre/post-treatment	Acupoints	Acupuncture type	Parameter/manipulation	Course	Results of molecular expression	Mechanisms of antagonizing oxidative stress
Zhang et al. [71]	Wistar rats Microemboli injection	Posttreatment	CV6 CV12 CV17 ST36 SP10	MA	Rotating the acupuncture needles at a frequency of 2-3 times per second clockwise for 30 s, respectively, once daily	21 sessions, commenced at the 13th day after surgery	MDA, O_2_^−^, GSSG↓, total SOD, CuZn-SOD, Mn-SOD, GSH↑	Preserving mitochondrial function and activation of mitochondrial antioxidative defense system
Liu et al. [69]	Wistar rats Microemboli injection	Posttreatment	CV17 CV12 CV6 ST36 SP10	MA	Twirling the acupuncture needles at a frequency of twice per second for 30 s at each point, once daily (1 day rest after 6 days of treatment)	18 sessions, commenced at the 8th day after surgery	Ref-1↑	Increasing the Ref-1 expression and exerting the antioxidant effects
Liu et al. [70]	Wistar rats Microemboli injection	Posttreatment	CV17 CV12 CV6 ST36 SP10	MA	Twirling the acupuncture needles at a frequency of twice per second for 30 s at each point, once daily (1 day rest after 6 days of treatment)	18 sessions, commenced at the 13th day after surgery	CuZn-SOD, GSH-Px↑	Upregulation of antioxidant factors
Siu et al. [60]	SD rats MCAO/R	Posttreatment	GB20	EA	2 Hz 0.7 V 30 min	One session, after surgery	MDA, 4-HNE↓, SOD, GSH-Px↑	Upregulation of antioxidant factors and regulating the lipid peroxidation
Shen et al. [97]	SD rats MCAO/R	Posttreatment	GV20 GV14	EA	3 Hz 1-3 mA 30 min	One session, 2 h after surgery	Nrf2↑	Increasing the Nrf2 expression and exerting the antioxidant effects
Shen et al. [96]	SD rats MCAO/R	Posttreatment	GV20 GV14	EA	3 Hz 1-3 mA 30 min	One session, commenced immediately after surgery	GCS, GCS subunits (GCSh and GCSl)↑	Upregulation of antioxidant factors
Cai et al. [63]	SD rats MCAO/R	Posttreatment	GV20 GV24	EA	2 Hz 1-3 mA 30 min/day	20 sessions, 5 sessions per week for 4 weeks after surgery	GSH↑	Upregulation of antioxidant factors
Lin et al. [64]	SD rats MCAO/R	Posttreatment	GV20 GV24	EA	5/20 Hz 1-3 mA 30 min/day	7 sessions, 7 consecutive days after surgery	MDA↓, SOD, GSH-Px↑	Upregulation of antioxidant factors and regulating the lipid peroxidation
Lin et al. [65]	SD rats MCAO/R	Posttreatment	GV20 GV24	EA	1-20 Hz 3-5 V 30 min/day	7 sessions, commenced at the 2nd day after surgery	MDA↓, SOD, GSH-Px↑	Upregulation of antioxidant factors and regulating the lipid peroxidation
Wang et al. [66]	SD rats MCAO/R	Posttreatment	GV20 GV26	EA	5/20 Hz 1-4 mA	One session, commenced immediately after surgery	MDA↓, SOD, GSH-Px↑	Upregulation of antioxidant factors and regulating the lipid peroxidation
Jittiwat et al. [67]	Wistar rats MCAO	Posttreatment	GV20	Ginger pharma acupuncture	Injecting a dose of 0.1 ml/kg ginger extract into the acupoint, once daily	14 sessions, 14 consecutive days after surgery	MDA↓, catalase, SOD, GSH-Px↑	Upregulation of antioxidant factors and regulating the lipid peroxidation
Jittiwat et al. [68]	Wistar rats MCAO	Posttreatment	GV20	Laser acupuncture	10 min/day, once daily	14 sessions, 14 consecutive days after surgery	MDA↓, catalase, SOD, GSH-Px↑	Upregulation of antioxidant factors and regulating the lipid peroxidation

EA: electroacupuncture; GCS: glutamylcysteine synthetase; GCSh: gamma-glutamylcysteine synthetase heavy subunit; GCSl: gamma-glutamylcysteine synthetase light subunit; GSH: glutathione; GSH-Px: glutathione peroxidase; GSSG: oxidized glutathione; 4-HNE: 4-hydroxynonenal; MA: manual acupuncture; MCAO/R: middle cerebral artery occlusion/reperfusion; MDA: malondialdehyde; Nrf2: nuclear factor erythroid-2-related factor 2; SD: Sprague-Dawley; SOD: superoxide dismutase; Ref: redox effector factor.

**Table 2:** Acupuncture inhibits the excessive generation of ROS/RNS.

References	Species/model	Pre/posttreatment	Acupoints	Acupuncture type	Parameter/manipulation	Course	Results of molecular expression	Mechanisms of antagonizing oxidative stress
Su et al. [78]	SD rats MCAO/R	Posttreatment	GV20 GV14 GV26 GV16	MA	20 min/day	15 sessions, commenced at the 11th day after surgery	NO, iNOS, O_2_^−^↓, SOD↑	Downregulation of pronitro/oxidative factors and upregulation of antioxidant factors
Shi et al. [74]	Wistar rats BCCAO	Posttreatment	GV20 ST36	MA	Twirling reinforcing manipulation at a frequency of more than twice per second for 30 s at each point, once daily (1 day rest after 6 days of treatment)	12 sessions, commenced at the 3rd day after surgery	O_2_^−^, NOX, NOX subunits (gp91phox and p47phox)↓	Inhibition of NOX-mediated ROS generation
Zhong et al. [80]	SD rats MCAO/R	Posttreatment	GV20 GV26	EA	5/20 Hz 2–4 mA 1 h	One session, commenced immediately after surgery	NADH dehydrogenase, succinic dehydrogenase, cytochrome C oxidase↑	Promoting the activities of respiratory enzymes and reducing the generation of ROS
Wang et al. [77]	SD rats MCAO/R	Posttreatment	GV20 ST36	EA	20 Hz 1 mA 30 min	One session, 2 h after surgery	NOX, ROS, MDA, ONOO^−^↓, SOD↑	Downregulation of pronitro/oxidative factors and upregulation of antioxidant factors
Jung et al. [28]	C57BL/6 J MCAO/R	Pretreatment	GV20 GV14	EA	2 Hz 1 mA 20 min/day	3 sessions, 3 successive days before surgery	O_2_^−^, NOX4↓	Reducing ROS generation with downregulation of NOX4
Guo et al. [75]	C57/BL6J MCAO/R with diabetes	Pretreatment	GV20	EA	2/15 Hz 1 mA 30 min	One session, 2 h before surgery	MDA, ROS, NOX subunits (gp91phox and p47phox)↓	Inhibition of NOX-mediated oxidative stress

BCCAO: bilateral common carotid artery occlusion; EA: electroacupuncture; iNOS: inducible nitric oxide synthase; MA: manual acupuncture; MCAO/R: middle cerebral artery occlusion/reperfusion; MDA: malondialdehyde; NO: nitric oxide; NOX: NADPH oxidase; SD: Sprague-Dawley; SOD: superoxide dismutase; ROS: reactive oxygen species.

**Table 3:** Acupuncture protects proteins and lipids from oxidative damage.

References	Species/model	Pre/post-treatment	Acupoints	Acupuncture type	Parameter/manipulation	Course	Results of molecular expression	Mechanisms of antagonizing oxidative stress
Siu et al. [83]	SD rats MCAO/R	Posttreatment	GB20/ST36	EA	2 Hz 0.7 V 30 min	One session, after surgery	Intact IgG molecules, Trx↑	Activation of Trx system and reducing the ROS-induced oxidative modifications of susceptible proteins
Siu et al. [61]	SD rats MCAO/R	Pretreatment	GB20/ST36	EA	2 Hz 0.7 V 30 min	One session, before surgery	MDA↓	Regulating the lipid peroxidation

EA: electroacupuncture; IgG: immunoglobulin G; MCAO/R: middle cerebral artery occlusion/reperfusion; MDA: malondialdehyde; SD: Sprague-Dawley; ROS: reactive oxygen species; Trx: thioredoxin.

**Table 4:** Acupuncture regulates the signaling pathways involved in redox modulation.

References	Species/model	Pre/posttreatment	Acupoints	Acupuncture type	Parameter/manipulation	Course	Results of molecular expression	Mechanisms of antagonizing oxidative stress
Yang et al. [27]	Wistar rats Microemboli injection	Posttreatment	ST36	MA	Twirling reinforcing manipulation at a frequency of more than twice per second for 30 s at each point, once daily (1 day rest after 6 days of treatment)	12 sessions, commenced at the 3rd day after surgery	·oh↓	Inhibiting activation of NF-*κ*B/p53 oxidative stress
Cheng et al. [79]	SD rats MCAO/R	Posttreatment	GV20 GV14	EA	5 Hz 2.7-3 mA 25 min/day	6 sessions, 6 consecutive days after surgery	iNOS↓	Downregulation of astrocytic S100B expression and inhibiting the p38 MAPK/NF-*κ*B/iNOS signaling pathway
Jin et al. [92]	SD rats MCAO/R	Posttreatment	GV20 GV14	EA	3 Hz 1–3 mA 30 min	One session, 2 h after surgery	GR, GSH, GSH-Px, GCS subunits (GCSh and GCSl)↑	Activation of the ERK1/2/Nrf2/GCS antioxidant signaling pathway
Fang et al. [95]	C57BL6 mice MCAO/R	Posttreatment	GV20 GV14	EA	2/15 Hz 1-3 mA 15 min	One session, 2 h after surgery	GSH, GSH/GSSG↑, GSSG↓	Activation of the Nrf2/GCS antioxidant signaling pathway
Chi et al. [62]	SD rats MCAO/R	Posttreatment	GV20 GV14	EA	2/15 Hz 1 mA 30 min	One session, 1 h after surgery	MDA↓	Activation of parasympathetic nervous system and muscarinic-mediated pathway
Long et al. [26]	SD rats MCAO/R	Pretreatment	GV20 BL23 SP6	EA	2/100 Hz 1 mA 1 h	One session, before surgery	MDA, cytochrome C↓, GSH, SOD↑	Inhibition of TRPV-1/p38 MAPK-mediated oxidative stress
Sun et al. [90]	C57BL/6 MCAO/R	Pretreatment	GV20	EA	2/15 Hz 1 mA 30 min	One session, 2 h before surgery	Mn-SOD↑, O_2_^−^↓	Activation of the CB1R-dependent STAT3/Mn-SOD signaling pathway
Shen et al. [94]	SD rats MCAO/R	Pre/posttreatment	GV20 GV14	EA	EA pretreatment: 2/15 Hz 1-3 mA 30 min/day EA posttreatment: 2/15 Hz 1-3 mA 30 min	EA pretreatment: 5 sessions, 5 consecutive days before surgery EA posttreatment: one session, commenced immediately after surgery	GSH, GSH-Px, GCS subunits (GCSh and GCSl)↑	Activation of the Nrf2/GCS antioxidant signaling pathway

CB1R: cannabinoid receptor type 1; EA: electroacupuncture; ERK1/2: extracellular regulated kinase 1/2; GCS: glutamylcysteine synthetase; GCSh: gamma-glutamylcysteine synthetase heavy subunit; GCSl: gamma-glutamylcysteine synthetase light subunit; GR: glutathione reductase; GSH: glutathione; GSH-Px: glutathione peroxidase; GSSG: oxidized glutathione; iNOS: inducible nitric oxide synthase; MA: manual acupuncture; MAPK: mitogen-activated protein kinase; MCAO/R: middle cerebral artery occlusion/reperfusion; MDA: malondialdehyde; NF-*κ*B: nuclear factor-*κ*B; Nrf2: nuclear factor erythroid-2-related factor 2; STAT3: signal transducer and activator of transcription 3; SD: Sprague-Dawley; SOD: superoxide dismutase; TRPV-1: transient receptor potential vanilloid 1.

**Table 5:** Frequency summary of individual acupoint appeared within the reviewed literatures.

Acupoint	Frequency of appearance
Baihui (GV20)	21
Dazhui (GV14)	9
Zusanli (ST36)	8
Fengchi (GB20)	3
Zhongwan (CV12)	3
Tanzhong (CV17)	3
Qihai (CV6)	3
Xuehai (SP10)	3
Shuigou (GV26)	3
Shenting (GV24)	3
Fengfu (GV16)	1
Sanyinjiao (SP6)	1
Shenshu (BL23)	1

## References

[1] GBD 2016 Stroke Collaborators (2019). Global, regional, and national burden of stroke, 1990-2016: a systematic analysis for the Global Burden of Disease Study 2016. *Lancet Neurology*.

[2] Stinear C. M., Lang C. E., Zeiler S., Byblow W. D. (2020). Advances and challenges in stroke rehabilitation. *Lancet Neurology*.

[3] Phipps M. S., Cronin C. A. (2020). Management of acute ischemic stroke. *British Medical Journal*.

[4] The GBD 2016 Lifetime Risk of Stroke Collaborators (2018). Global, regional, and country-specific lifetime risks of stroke, 1990 and 2016. *New England Journal of Medicine*.

[5] Benjamin E. J., Muntner P., Alonso A. (2019). Heart disease and stroke Statistics-2019 update: a report from the American Heart Association. *Circulation*.

[6] Campbell B. C. V., de Silva D. A., Macleod M. R. (2019). Ischaemic stroke. *Nature Reviews Disease Primers*.

[7] Powers W. J., Rabinstein A. A., Ackerson T. (2018). 2018 guidelines for the early management of patients with acute ischemic stroke: a guideline for healthcare professionals from the American Heart Association/American Stroke Association. *Stroke*.

[8] Turc G., Bhogal P., Fischer U. (2019). European Stroke Organisation (ESO) - European Society for Minimally Invasive Neurological Therapy (ESMINT) Guidelines on mechanical thrombectomy in acute ischemic stroke. *Journal of NeuroInterventional Surgery*.

[9] Menon B. K., al-Ajlan F. S., Najm M. (2018). Association of clinical, imaging, and thrombus characteristics with recanalization of visible intracranial occlusion in patients with acute ischemic stroke. *Journal of the American Medical Association*.

[10] Baron J. C. (2018). Protecting the ischaemic penumbra as an adjunct to thrombectomy for acute stroke. *Nature Reviews Neurology*.

[11] Kahles T., Brandes R. P. (2012). NADPH oxidases as therapeutic targets in ischemic stroke. *Cellular and Molecular Life Sciences*.

[12] Chen H., Yoshioka H., Kim G. S. (2011). Oxidative stress in ischemic brain damage: mechanisms of cell death and potential molecular targets for neuroprotection. *Antioxidants & Redox Signaling*.

[13] Allen C. L., Bayraktutan U. (2009). Oxidative stress and its role in the pathogenesis of ischaemic stroke. *International Journal of Stroke*.

[14] Nathan C., Ding A. (2010). SnapShot: reactive oxygen intermediates (ROI). *Cell*.

[15] Kahles T., Luedike P., Endres M. (2007). NADPH oxidase plays a central role in blood-brain barrier damage in experimental stroke. *Stroke*.

[16] Casas A. I., Geuss E., Kleikers P. W. M. (2017). NOX4-dependent neuronal autotoxicity and BBB breakdown explain the superior sensitivity of the brain to ischemic damage. *Proceedings of the National Academy of Sciences of the United States of America*.

[17] Canty T. G., Boyle E. M., Farr A., Morgan E. N., Verrier E. D., Pohlman T. H. (1999). Oxidative stress induces NF-*κ*B nuclear translocation without degradation of I*κ*B*α*. *Circulation*.

[18] Kleinschnitz C., Grund H., Wingler K. (2010). Post-stroke inhibition of induced NADPH oxidase type 4 prevents oxidative stress and neurodegeneration. *PLoS Biology*.

[19] Forrester S. J., Kikuchi D. S., Hernandes M. S., Xu Q., Griendling K. K. (2018). Reactive oxygen species in metabolic and inflammatory signaling. *Circulation Research*.

[20] Davies A. M., Holt A. G. (2018). Why antioxidant therapies have failed in clinical trials. *Journal of Theoretical Biology*.

[21] Bjelakovic G., Nikolova D., Gluud L. L., Simonetti R. G., Gluud C. (2007). Mortality in randomized trials of antioxidant supplements for primary and secondary prevention: systematic review and meta-analysis. *Journal of the American Medical Association*.

[22] Xu M., Li D., Zhang S. (2018). Acupuncture for acute stroke. *Cochrane Database of Systematic Reviews*.

[23] Zhang S., Wu B., Liu M. (2015). Acupuncture efficacy on ischemic stroke recovery. *Stroke*.

[24] Wang W. W., Xie C. L., Lu L., Zheng G. Q. (2014). A systematic review and meta-analysis of Baihui (GV20)-based scalp acupuncture in experimental ischemic stroke. *Scientific Reports*.

[25] Tanaka L. Y., Laurindo F. R. M. (2018). The eye of the needle: redox mechanisms of acupuncture effects in hypertension. *Hypertension*.

[26] Long M., Wang Z., Zheng D. (2019). Electroacupuncture pretreatment elicits neuroprotection against cerebral ischemia-reperfusion injury in rats associated with transient receptor potential vanilloid 1-mediated anti-oxidant stress and anti-inflammation. *Inflammation*.

[27] Yang J. W., Wang X. R., Ma S. M., Yang N. N., Li Q. Q., Liu C. Z. (2019). Acupuncture attenuates cognitive impairment, oxidative stress and NF-*κ*B activation in cerebral multi-infarct rats. *Acupuncture in Medicine*.

[28] Jung Y. S., Lee S. W., Park J. H., Seo H. B., Choi B. T., Shin H. K. (2016). Electroacupuncture preconditioning reduces ROS generation with NOX4 down-regulation and ameliorates blood-brain barrier disruption after ischemic stroke. *Journal of Biomedical Science*.

[29] Lyle A. N., Griendling K. K. (2006). Modulation of vascular smooth muscle signaling by reactive oxygen species. *Physiology*.

[30] Droge W. (2002). Free radicals in the physiological control of cell function. *Physiological Reviews*.

[31] Chan P. H. (1996). Role of oxidants in ischemic brain damage. *Stroke*.

[32] Briones A. M., Touyz R. M. (2010). Oxidative stress and hypertension: current concepts. *Current Hypertension Reports*.

[33] Saeed S. A., Shad K. F., Saleem T., Javed F., Khan M. U. (2007). Some new prospects in the understanding of the molecular basis of the pathogenesis of stroke. *Experimental Brain Research*.

[34] Chrissobolis S., Faraci F. M. (2008). The role of oxidative stress and NADPH oxidase in cerebrovascular disease. *Trends in Molecular Medicine*.

[35] Olmez I., Ozyurt H. (2012). Reactive oxygen species and ischemic cerebrovascular disease. *Neurochemistry International*.

[36] Dworakowski R., Alom-Ruiz S. P., Shah A. M. (2008). NADPH oxidase-derived reactive oxygen species in the regulation of endothelial phenotype. *Pharmacological Reports*.

[37] Fukai T., Ushio-Fukai M. (2011). Superoxide dismutases: role in redox signaling, vascular function, and diseases. *Antioxidants & Redox Signaling*.

[38] Ridnour L. A., Thomas D. D., Mancardi D. (2004). The chemistry of nitrosative stress induced by nitric oxide and reactive nitrogen oxide species. Putting perspective on stressful biological situations. *Biological Chemistry*.

[39] Ribas V., Garcia-Ruiz C., Fernandez-Checa J. C. (2014). Glutathione and mitochondria. *Frontiers in Pharmacology*.

[40] Bergendi L., Beneš L., Ďuračková Z., Ferenčik M. (1999). Chemistry, physiology and pathology of free radicals. *Life Sciences*.

[41] Chance B., Sies H., Boveris A. (1979). Hydroperoxide metabolism in mammalian organs. *Physiological Reviews*.

[42] Beckman J. S., Koppenol W. H. (1996). Nitric oxide, superoxide, and peroxynitrite: the good, the bad, and ugly. *American Journal of Physiology*.

[43] Pacher P., Beckman J. S., Liaudet L. (2007). Nitric oxide and peroxynitrite in health and disease. *Physiological Reviews*.

[44] Stamler J. S., Simon D. I., Osborne J. A. (1992). S-nitrosylation of proteins with nitric oxide: synthesis and characterization of biologically active compounds. *Proceedings of the National Academy of Sciences of the United States of America*.

[45] Radi R. (1996). Reactions of nitric oxide with metalloproteins. *Chemical Research in Toxicology*.

[46] Vásquez-Vivar J., Kalyanaraman B., Martásek P. (1998). Superoxide generation by endothelial nitric oxide synthase: the influence of cofactors. *Proceedings of the National Academy of Sciences of the United States of America*.

[47] Thomas S. R., Witting P. K., Drummond G. R. (2008). Redox control of endothelial function and dysfunction: molecular mechanisms and therapeutic opportunities. *Antioxidants & Redox Signaling*.

[48] Jones D. P. (2008). Radical-free biology of oxidative stress. *American Journal of Physiology—Cell Physiology*.

[49] Deb P., Sharma S., Hassan K. M. (2010). Pathophysiologic mechanisms of acute ischemic stroke: an overview with emphasis on therapeutic significance beyond thrombolysis. *Pathophysiology*.

[50] Sanderson T. H., Reynolds C. A., Kumar R., Przyklenk K., Huttemann M. (2013). Molecular mechanisms of ischemia-reperfusion injury in brain: pivotal role of the mitochondrial membrane potential in reactive oxygen species generation. *Molecular Neurobiology*.

[51] Domínguez C., Delgado P., Vilches A. (2010). Oxidative stress after thrombolysis-induced reperfusion in human stroke. *Stroke*.

[52] Cojocaru I. M., Cojocaru M., Sapira V., Ionescu A. (2013). Evaluation of oxidative stress in patients with acute ischemic stroke. *Romanian Journal of Internal Medicine*.

[53] Yang G., Chan P. H., Chen J. (1994). Human copper-zinc superoxide dismutase transgenic mice are highly resistant to reperfusion injury after focal cerebral ischemia. *Stroke*.

[54] Alexander A., Cai S. L., Kim J. (2010). ATM signals to TSC2 in the cytoplasm to regulate mTORC1 in response to ROS. *Proceedings of the National Academy of Sciences of the United States of America*.

[55] Crack P. J., Taylor J. M. (2005). Reactive oxygen species and the modulation of stroke. *Free Radical Biology & Medicine*.

[56] Gursoy-Ozdemir Y., Can A., Dalkara T. (2004). Reperfusion-induced oxidative/nitrative injury to neurovascular unit after focal cerebral ischemia. *Stroke*.

[57] Usatyuk P. V., Natarajan V. (2005). Regulation of reactive oxygen species-induced endothelial cell-cell and cell-matrix contacts by focal adhesion kinase and adherens junction proteins. *American Journal of Physiology—Lung Cellular and Molecular Physiology*.

[58] Bektas H., Wu T. C., Kasam M. (2010). Increased blood-brain barrier permeability on perfusion CT might predict malignant middle cerebral artery infarction. *Stroke*.

[59] Kahles T., Kohnen A., Heumueller S. (2010). NADPH oxidase Nox1 contributes to ischemic injury in experimental stroke in mice. *Neurobiology of Disease*.

[60] Siu F. K. W., Lo S. C. L., Leung M. C. P. (2004). Electroacupuncture reduces the extent of lipid peroxidation by increasing superoxide dismutase and glutathione peroxidase activities in ischemic-reperfused rat brains. *Neuroscience Letters*.

[61] Siu F. K. W., Lo S. C. L., Leung M. C. P. (2004). Effectiveness of multiple pre-ischemia electro-acupuncture on attenuating lipid peroxidation induced by cerebral ischemia in adult rats. *Life Sciences*.

[62] Chi L., Du K., Liu D., Bo Y., Li W. (2018). Electroacupuncture brain protection during ischemic stroke: a role for the parasympathetic nervous system. *Journal of Cerebral Blood Flow & Metabolism*.

[63] Cai W., Ma W., Wang G. T., Li Y. J., Shen W. D. (2019). Antidepressant, anti-inflammatory, and antioxidant effects of electroacupuncture through sonic hedgehog-signaling pathway in a rat model of poststroke depression. *Neuropsychiatric Disease and Treatment*.

[64] Lin R., Lin Y., Tao J. (2015). Electroacupuncture ameliorates learning and memory in rats with cerebral ischemia-reperfusion injury by inhibiting oxidative stress and promoting p-CREB expression in the hippocampus. *Molecular Medicine Reports*.

[65] Lin Y. K., Lin R. H., Chen B., Yu K. Q., Tao J. (2015). The possible mechanism of electroacupuncture ameliorating learning and memory ability in rats with focal cerebral ischemia/reperfusion via inhibiting oxidative stress. *Chinese Journal of Rehabilitation Medicine*.

[66] Wang C. X., Li Z. R., Chen B. Y. (2005). Protective effect of electroacupuncture on cerebral function via ameliorating oxidative stress in MCAO rats. *Neuroscience Bulletin*.

[67] Jittiwat J., Wattanathorn J. (2012). Ginger pharmacopuncture improves cognitive impairment and oxidative stress following cerebral ischemia. *Journal of Acupuncture and Meridian Studies*.

[68] Jittiwat J. (2017). Laser acupuncture at GV20 improves brain damage and oxidative stress in animal model of focal ischemic stroke. *Journal of Acupuncture and Meridian Studies*.

[69] Liu C. Z., Li Z. G., Wang D. J. (2013). Effect of acupuncture on hippocampal Ref-1 expression in cerebral multi-infarction rats. *Neurological Sciences*.

[70] Liu C. Z., Yu J. C., Zhang X. Z., Fu W. W., Wang T., Han J. X. (2006). Acupuncture prevents cognitive deficits and oxidative stress in cerebral multi-infarction rats. *Neuroscience Letters*.

[71] Zhang X., Wu B., Nie K., Jia Y., Yu J. (2014). Effects of acupuncture on declined cerebral blood flow, impaired mitochondrial respiratory function and oxidative stress in multi-infarct dementia rats. *Neurochemistry International*.

[72] Feng D., Yang C., Geurts A. M. (2012). Increased expression of NAD(P)H oxidase subunit p67(phox) in the renal medulla contributes to excess oxidative stress and salt-sensitive hypertension. *Cell Metabolism*.

[73] Infanger D. W., Sharma R. V., Davisson R. L. (2006). NADPH oxidases of the brain: distribution, regulation, and function. *Antioxidants & Redox Signaling*.

[74] Shi G. X., Wang X. R., Yan C. Q. (2015). Acupuncture elicits neuroprotective effect by inhibiting NAPDH oxidase-mediated reactive oxygen species production in cerebral ischaemia. *Scientific Reports*.

[75] Guo F., Song W., Jiang T. (2014). Electroacupuncture pretreatment inhibits NADPH oxidase-mediated oxidative stress in diabetic mice with cerebral ischemia. *Brain Research*.

[76] Garry P. S., Ezra M., Rowland M. J., Westbrook J., Pattinson K. T. S. (2015). The role of the nitric oxide pathway in brain injury and its treatment--from bench to bedside. *Experimental Neurology*.

[77] Wang H., Chen S., Zhang Y., Xu H., Sun H. (2019). Electroacupuncture ameliorates neuronal injury by Pink1/Parkin-mediated mitophagy clearance in cerebral ischemia-reperfusion. *Nitric Oxide*.

[78] Su X., Wu Z., Mai F. (2019). ‘Governor vessel-unblocking and mind-regulating’ acupuncture therapy ameliorates cognitive dysfunction in a rat model of middle cerebral artery occlusion. *International Journal of Molecular Medicine*.

[79] Cheng C. Y., Lin J. G., Tang N. Y., Kao S. T., Hsieh C. L. (2014). Electroacupuncture-like stimulation at the Baihui (GV20) and Dazhui (GV14) acupoints protects rats against subacute-phase cerebral ischemia-reperfusion injuries by reducing S100B-mediated neurotoxicity. *PLoS One*.

[80] Zhong S., Li Z., Huan L., Chen B. Y. (2009). Neurochemical mechanism of electroacupuncture: anti-injury effect on cerebral function after focal cerebral ischemia in rats. *Evidence-based Complementary and Alternative Medicine*.

[81] Stadtman E. R., Berlett B. S. (1998). Reactive oxygen-mediated protein oxidation in aging and disease. *Drug Metabolism Reviews*.

[82] Nakamura H. (2004). Thioredoxin as a key molecule in redox signaling. *Antioxidants & Redox Signaling*.

[83] Siu F. K. W., Lo S. C. L., Leung M. C. P. (2005). Electro-acupuncture potentiates the disulphide-reducing activities of thioredoxin system by increasing thioredoxin expression in ischemia-reperfused rat brains. *Life Sciences*.

[84] Nordberg J., Arner E. S. J. (2001). Reactive oxygen species, antioxidants, and the mammalian thioredoxin system. *Free Radical Biology & Medicine*.

[85] Liu F., Lu J., Manaenko A., Tang J., Hu Q. (2018). Mitochondria in ischemic stroke: new insight and implications. *Aging and Disease*.

[86] Narendra D. P., Jin S. M., Tanaka A. (2010). PINK1 is selectively stabilized on impaired mitochondria to activate Parkin. *PLoS Biology*.

[87] Cheyuo C., Jacob A., Wu R., Zhou M., Coppa G. F., Wang P. (2011). The parasympathetic nervous system in the quest for stroke therapeutics. *Journal of Cerebral Blood Flow & Metabolism*.

[88] Miao Y., Zhou J., Zhao M. (2013). Acetylcholine attenuates hypoxia/ reoxygenation-induced mitochondrial and cytosolic ROS formation in H9c2 cells via M2 acetylcholine receptor. *Cellular Physiology and Biochemistry*.

[89] Jung J. E., Kim G. S., Narasimhan P., Song Y. S., Chan P. H. (2009). Regulation of Mn-superoxide dismutase activity and neuroprotection by STAT3 in mice after cerebral ischemia. *Journal of Neuroscience*.

[90] Sun S., Chen X., Gao Y. (2016). Mn-SOD upregulation by electroacupuncture attenuates ischemic oxidative damage via CB1R-mediated STAT3 phosphorylation. *Molecular Neurobiology*.

[91] Ma W. W., Li C. Q., Yu H. L. (2015). The oxysterol 27-hydroxycholesterol increases oxidative stress and regulate Nrf2 signaling pathway in astrocyte cells. *Neurochemical Research*.

[92] Jin X. L., Li P. F., Zhang C. B. (2016). Electroacupuncture alleviates cerebral ischemia and reperfusion injury via modulation of the ERK1/2 signaling pathway. *Neural Regeneration Research*.

[93] Mulcahy R. T., Wartman M. A., Bailey H. H., Gipp J. J. (1997). Constitutive and beta-naphthoflavone-induced expression of the human gamma-glutamylcysteine synthetase heavy subunit gene is regulated by a distal antioxidant response element/TRE sequence. *Journal of Biological Chemistry*.

[94] Shen M. H., Zhang C. B., Zhang J. H., Li P. F. (2016). Electroacupuncture attenuates cerebral ischemia and reperfusion injury in middle cerebral artery occlusion of rat via modulation of apoptosis, inflammation, oxidative stress, and excitotoxicity. *Evidence-based Complementary and Alternative Medicine*.

[95] Fang C. C., Jin X. L., Xu Q. Q., Shen J., Li Q., Shen M. H. (2019). Regulation mechanism of electroacupuncture on apoptosis in mice with cerebral ischemia-reperfusion injury based on Nrf2 signaling pathway. *Chinese Journal of Integrative Medicine on Cardio-Cerebrovascular Disease*.

[96] Shen M. H., Xiang X. R., Li Y., Pan J. L., Ma C., Li Z. R. (2012). Effect of electroacupuncture on expression of gamma-glutamylcysteine synthetase protein and mRNA in cerebral cortex in rats with focal cerebral ischemia-reperfusion. *Zhen Ci Yan Jiu*.

[97] Shen M. H., Shen J., Shu Z. R., Zhang Y., Mu Y. Y., Li Z. R. (2014). Effect of electro-acupuncture on the expression of Nrf2 protein in cerebral cortex of cerebral ischemia reperfusion rat model. *Chinese Journal of Gerontology*.

[98] Shi G. X., Yang X. M., Liu C. Z., Wang L. P. (2012). Factors contributing to therapeutic effects evaluated in acupuncture clinical trials. *Trials*.

[99] Fang Z., Ning J., Xiong C., Shulin Y. (2012). Effects of electroacupuncture at head points on the function of cerebral motor areas in stroke patients: a PET study. *Evidence-based Complementary and Alternative Medicine*.

[100] Wang Q., Wang F., Li X. (2012). Electroacupuncture pretreatment attenuates cerebral ischemic injury through *α*7 nicotinic acetylcholine receptor-mediated inhibition of high-mobility group box 1 release in rats. *Journal of Neuroinflammation*.

[101] Kim J. H., Choi K. H., Jang Y. J. (2013). Electroacupuncture acutely improves cerebral blood flow and attenuates moderate ischemic injury via an endothelial mechanism in mice. *PLoS One*.

[102] Cai W., Shen W. D. (2018). Anti-apoptotic mechanisms of acupuncture in neurological diseases: a review. *American Journal of Chinese Medicine*.

[103] Kim Y. R., Kim H. N., Ahn S. M., Choi Y. H., Shin H. K., Choi B. T. (2014). Electroacupuncture promotes post-stroke functional recovery via enhancing endogenous neurogenesis in mouse focal cerebral ischemia. *PLoS One*.

[104] Wu C., Wang J., Li C. (2015). Effect of electroacupuncture on cell apoptosis and ERK signal pathway in the hippocampus of adult rats with cerebral ischemia-reperfusion. *Evidence-Based Complementary and Alternative Medicine*.

[105] Lan L., Tao J., Chen A. (2012). Electroacupuncture exerts anti-inflammatory effects in cerebral ischemia-reperfusion injured rats via suppression of the TLR4/NF-*κ*B pathway. *International Journal of Molecular Medicine*.

[106] NIN Consensus Development Panel on Acupuncture (1998). NIH consensus conference. Acupuncture. *Journal of the American Medical Association*.

[107] Shen B. (2015). A new golden age of natural products drug discovery. *Cell*.

[108] Yang X., He T., Han S. (2019). The role of traditional Chinese medicine in the regulation of oxidative stress in treating coronary heart disease. *Oxidative Medicine and Cellular Longevity*.

[109] Fisher M., Saver J. L. (2015). Future directions of acute ischaemic stroke therapy. *Lancet Neurology*.

